# Association between immune-related adverse events and prognosis in patients with advanced non-small cell lung cancer: a systematic review and meta-analysis

**DOI:** 10.3389/fonc.2024.1402017

**Published:** 2024-05-08

**Authors:** Shixin Ma, He Nie, Chaoyu Wei, Cailong Jin, Lunqing Wang

**Affiliations:** ^1^ Graduate School, Dalian Medical University, Dalian, Liaoning, China; ^2^ Department of Thoracic Surgery, Qingdao Municipal Hospital, Qingdao, Shandong, China; ^3^ Graduate School, Xi ‘an Medical University, Xi ‘an, Shanxi, China; ^4^ Department of Thoracic Surgery, Qingdao Women, And Children Hospital (Women and Children’s Hospital Affiliated to Qingdao University), Qingdao, China

**Keywords:** non-small-cell lung carcinoma, immune checkpoint inhibitors, immune-related adverse events, prognosis, meta - analysis

## Abstract

**Background:**

The emergence of immune checkpoint inhibitors (ICIs) provides a variety of options for patients with advanced non-small-cell lung cancer (NSCLC). After the application of ICIs, the immune system of patients was highly activated, and immune-related adverse events (irAEs) could occur in some organ systems, and irAEs seemed to be associated with the survival prognosis of patients. Therefore, we evaluated the association between survival outcomes and irAEs in NSCLC patients and conducted a systematic review and meta-analysis.

**Methods:**

We conducted systematic reviews of PubMed, Embase, Cochrane, and Web of Science databases until December 2021. The forest map was constructed by combining the hazard ratio (HR) and 95% confidence interval (CI). I^2^ estimated the heterogeneity between studies. A meta-analysis was performed using R 4.2.1 software.

**Results:**

Eighteen studies included 4808 patients with advanced NSCLC. In pooled analysis, the occurrence of irAEs was found to be a favorable factor for improved prognosis (PFS: HR: 0.48, 95% CI: 0.41-0.55, P <0.01; OS: HR: 0.46, 95% CI: 0.42-0.52, P <0.01). In subgroup analyses, cutaneous irAE, gastrointestinal irAE, endocrine irAE and grade ≥3 irAEs were associated with improvements in PFS and OS, but pulmonary and hepatic irAEs were not.

**Conclusion:**

Existing evidence suggests that the occurrence of irAEs may be a prognostic biomarker for advanced NSCLC. However, further research is needed to explore the prospect of irAEs as a prognostic biomarker in patients undergoing immunotherapy.

**Systematic review registration:**

https://www.crd.york.ac.uk/PROSPEROFILES/405333_STRATEGY_20240502.pdf, identifier CRD42023405333.

## Introduction

Lung cancer represents 11.4% of all malignancies and 18% of all cancer-related fatalities, making it the primary cause of mortality from cancer, according to Global Cancer Statistics 2020 (1). Non-small-cell lung cancer (NSCLC) comprises approximately 80–85% of all lung cancer cases and exhibits a poor 5-year survival rate (2). Patients with early NSCLC typically undergo surgery followed by adjuvant therapy to reduce the risk of cancer recurrence and enhance patient survival (3). With the progress of clinical diagnosis and treatment technology, the early detection rate of lung cancer has increased significantly, and the 5-year survival rate of patients has improved (4). However, some patients are diagnosed with advanced lung cancer and cannot benefit from surgery. The emergence of targeted therapy and immunotherapy provides a variety of options for lung cancer patients. ICIs relieve the suppression of immune function caused by immune checkpoints by blocking the binding of immune checkpoints with their ligands so as to reactivate immune cells to play an anti-tumor role (5). Tumor mutational burden (TMB) and programmed cell death-ligand 1 (PD-L1) expression are often utilized biomarkers for assessing therapy response and prognosis in patients. However, they are not considered the optimal biomarkers due to considerations including high cost, lengthy processing time, and inadequate tumor samples (6–8). After the application of ICIs, the immune system of patients is highly activated, and immune-related adverse events (irAEs) can occur in some organ systems, the most common of which are the cutaneous, gastrointestinal tract, endocrine system, liver, and lung. Others include nervous system, blood system, heart, eye, and rheumatic system involvement (9, 10). Previous studies have shown that the development of irAEs is associated with improved melanoma prognosis (11). The emergence or development of irAEs may be used as an alternative indicator to judge the efficacy of ICIs and evaluate the survival and prognosis of patients. This connection makes it crucial to monitor the adverse reactions after treatment with ICIs. However, the results of existing studies are not the same. Therefore, in order to strengthen the relationship between irAEs and the survival outcome of NSCLC patients, this study conducted a systematic review of the studies of patients with advanced NSCLC receiving immunotherapy and developing irAEs to investigate the relationship between irAEs and the survival prognosis of NSCLC patients.

## Materials and methods

### Literature retrieval strategy

We utilized the PICOS framework to formulate study questions and conduct literature searches. The participants were individuals diagnosed with lung cancer, namely NSCLC. The intervention was immune checkpoint inhibitor therapy, and the result was irAEs. We searched PubMed, Embase, Cochrane, and Web of Science databases for studies reporting irAEs and prognosis after ICIs in NSCLC patients from database creation until December 2021. Key search terms included lung cancer, non-small cell lung cancer, irAEs, immune checkpoint inhibitors, programmed death- 1 (PD-1) or PD-L1 inhibitors, and cytotoxic t lymphocyte-associated antigen-4 (CTLA-4) inhibitors, as well as those identified by the Food and Drug Administration. The food and drug administration (FDA) approved immune checkpoint inhibitor drug already on the market.

### Inclusion and exclusion criteria

The included studies met the following criteria: (1) prospective or retrospective studies to investigate the effect of irAEs on prognosis in patients with NSCLC; (2) have been clinically diagnosed with advanced non-small cell lung cancer and have been treated with at least one or more ICIs; (3) strictly in accordance with the definition of irAEs classification and clear grouping; (4) articles including hazard ratios (HR) and 95% confidence intervals (CI) for overall survival (OS) and progression-free survival (PFS). (5) Research published in English. The exclusion criteria were as follows: (1) The patient was known to have an autoimmune disease, and the adverse events reported in the study were not significantly associated with ICIs; (2) In order to avoid confusion about adverse reactions caused by other drugs, studies receiving immunotherapy in combination with other anti-tumor therapies, including combination chemotherapy, radiation therapy, targeted therapy, and antiangiogenic therapy, were excluded. (3) Studies without HR and 95% CI data. (4) Review articles, case reports, animal studies, and cost-benefit studies.

### Data collection and quality assessment

Two researchers are responsible for the first phase of independent screening of titles and abstracts and the second stage of full text screening, a full text review of all potentially relevant citations to determine the final inclusion of the study. If there are any unresolved differences, discuss them with the third researcher and resolve them. The extracted data included author, publication year, sample size, population of irAEs occurrence, irAEs type and grade, and OS and PFS of patients with and without irAEs. HR, 95% CI, and P-value were extracted from the Cox regression analysis and survival curve. According to the occurrence of irAEs, they were divided into an irAEs group and a non-irAEs group. In addition, HR provided by irAEs of any grade or organ is selected when HR of irAEs of any grade or organ, graded irAEs, and single organs is presented simultaneously in the study. When both univariate and multivariate HR are provided for any grade or any type of irAEs in the study, the HR provided in multivariate analysis is selected.

### Meta analysis

The primary goal of this study was to evaluate the association between OS, PFS, and irAEs in NSCLC receiving immunotherapy. The secondary objective was to evaluate the relationship between irAEs organ and irAEs grade with OS and PFS. Meta-analysis was performed using R4.2.1. The forest map was constructed by combining HR and 95% CI. Heterogeneity between studies was estimated by I^2^. If I^2^ >50% indicates significant heterogeneity, the meta-analysis uses a random effects model (12). Instead, a fixed effects model is used (13). P-values below 0.05 were considered statistically significant.

## Results

### Literature search results

**Figure 1:**
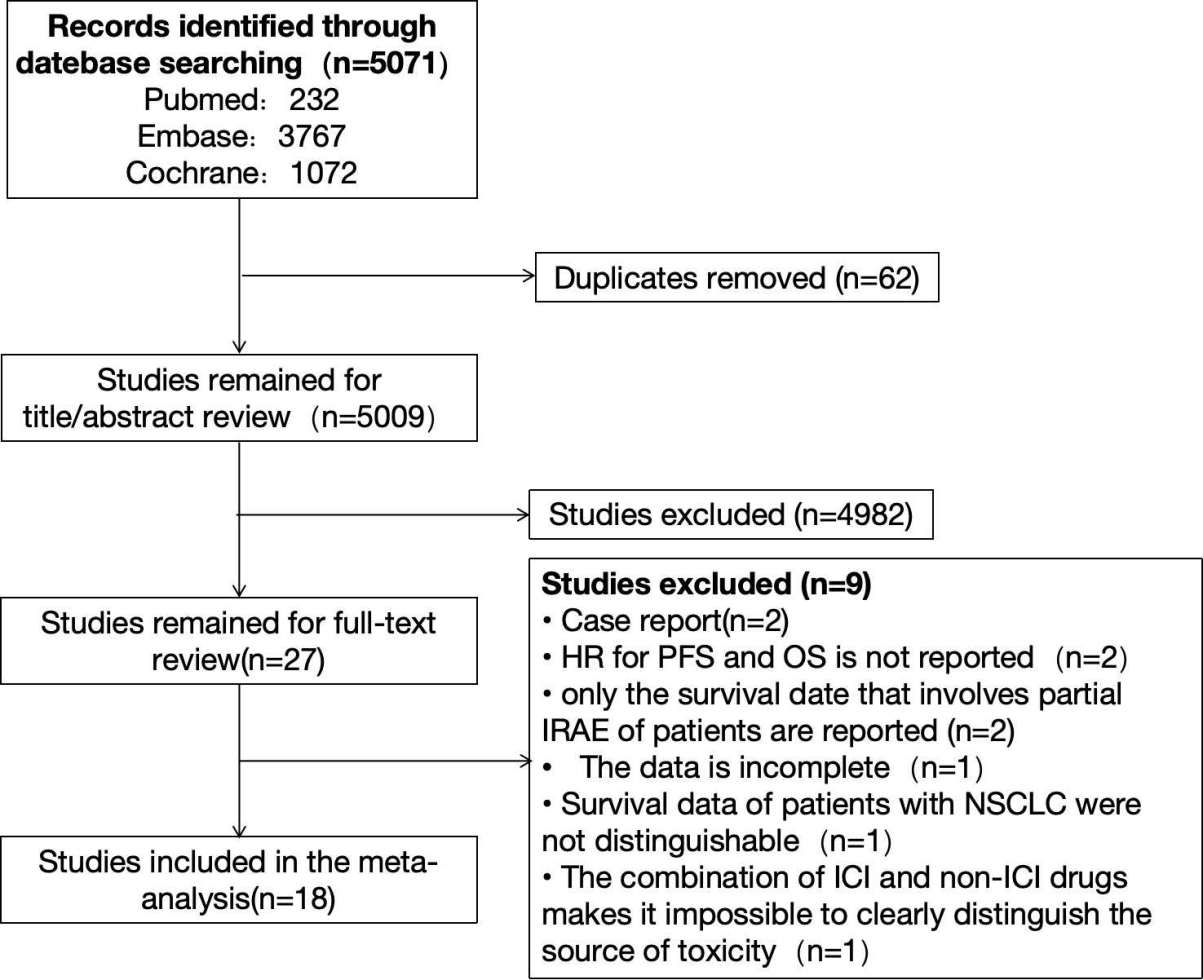
Flowchart and the detailed process of eligible studies.

We searched PubMed, Embase, and Cochrane databases, respectively. There were 232 citations found in PubMed, 3,767 citations found in Embase, and 1,072 citations found in Cochrane, for a total of 5,071 citations. After sifting through the titles and abstracts, we collected from them 27 studies that might qualify. Finally, after a full review of the articles, we selected 18 studies. The reasons for exclusion are as follows: Two reports had survival data but fell under the category of case reports. Two studies only reported OS and PFS data but did not report the corresponding HR. Two studies reported survival data only for irAE patients involving a single organ system; one study had incomplete data and could not be included in the analysis; and in one study, survival data of patients with other types of tumors were pooled. Survival data for patients with NSCLC were indistinguishable. The study was a combination of ICIs and non-ICIs drugs and radiation therapy and could not clearly distinguish the source of adverse events. The detailed retrieval process is shown in **Figure 1**. The meta-analysis included 18 studies of 4808 patients with advanced NSCLC, with a sample size ranging from 23 to 1010 patients. Sixteen studies were retrospective, and two were prospective (14–31). Main characteristics of the included studies as shown in **Table 1**.

**Table 1:** Main characteristics of the included studies.

Study	n	ICIs	irAEs(%)	PFS		OS	
				irAEs+	irAEs-	irAEs+	irAEs-
Ahn et al., 2019 (14)	155	Nivolumab	61.93%	11.63	3.27	24.05	7.39
		Pembrolizumab					
Akamatsu et al., 2020 (15)	23	Nivolumab	65.21%	19.10	5.60	27.80	16.10
		Pembrolizumab					
		Atezolizumab					
Chen et al., 2020 (16)	97	Pembrolizumab	46.39%	11.30	2.80	17.90	–
		Nivolumab					
Chen et al., 2021 (17)	191	PD-1/PD-L1	36.60%	8.80	3.90	21.00	14.80
Conde-Estévez et al., 2021 (18)	70	Nivolumab	44.30%	13.00	1.90	30.10	5.10
		Pembrolizumab					
		Atezolizumab					
Cortellini et al., 2019 (19)	559	Nivolumab	41.32%	10.10	4.10	20.50	8.50
		Pembrolizumab					
Cortellini et al., 2020 (20)	1010	Pembrolizumab	32.97%	19.90	7.80	–	16.10
Daniello et al., 2021 (21)	894	PD-1/PD-L1	22.10%	17.00	10.00	37.00	15.00
Grangeon et al., 2019 (22)	270	PD-1/PD-L1	45.92%	5.20	1.97	–	8.21
Haratani et al., 2018 (23)	134	Nivolumab	51.49%	9.20	4.80	not reached	11.10
Hosoya et al., 2020 (24)	76	Nivolumab	57.89%	4.00	1.90	not reached	13.00
K.Komiya et al., 2019 (25)	61	nivolumab	29.50%	9.30	1.90	not reached	8.70
		pembrolizumab					
Naqash et al., 2020 (26)	531	Nivolumab	32.58%	6.10	3.10	14.90	7.40
Noguchi et al., 2020 (27)	94	Pembrolizumab	67.02%	12.40	2.20	not reached	not reached
Ricciuti et al., 2019 (28)	195	Nivolumab	43.58%	5.70	2.00	17.80	4.00
Riudavets et al., 2020 (29)	267	PD-1/PD-L1	56.90%	12.40	4.10	28.20	12.50
Sonehara et al., 2022 (30)	80	Nivolumab	31.25%	6.80	1.90	37.80	8.10
		Pembrolizumab					
		Atezolizumab					
Y. Wu et al., 2022 (31)	101	PD-1/PD-L1	44.60%	7.00	4.00	17.00	9.00

ICIs, immune checkpoint inhibitor; irAEs, Immune-related adverse events; PFS, progression-free survival; OS, Overall survival; “-”, indicates data not reported in the original publication.

### The correlation between irAEs occurrence, PFS and OS

**Figure 2:**
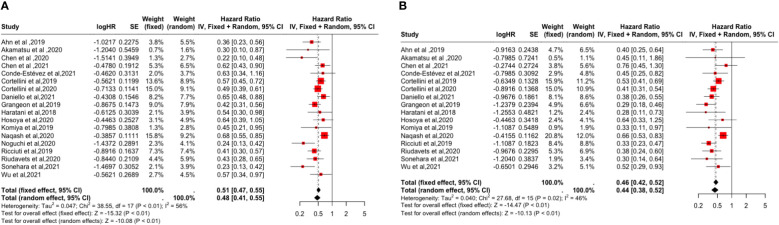
Forest plot of the association between the occurrence of irAEs and PFS and OS. **(A)** Progression-Free Survival (PFS); **(B)** Overall Survival (OS).

In the meta-analysis, 18 studies all provided HRs for PFS (14–31) and 16 studies evaluated HRs for OS (14, 15, 17–26, 28–31), and the pooled analysis showed that the occurrence of irAEs was a favorable factor for improvement in PFS and OS (PFS: [HR: 0.48, 95% CI: 0.41-0.55, P <0.01]; OS: [HR: 0.46, 95% CI: 0.42-0.52, P <0.01]. As shown in **Figure 2**, Synthetic analysis showed moderate heterogeneity between irAEs and OS studies (I^2 =^ 46%, P = 0.02) and significant heterogeneity between PFS studies (I^2 =^ 56%, P <0.01). The heterogeneity may be related to the organ and grade of irAEs. Therefore, we conducted a subgroup analysis of the correlation between the occurrence and prognosis of irAEs.

**Figure 3:**
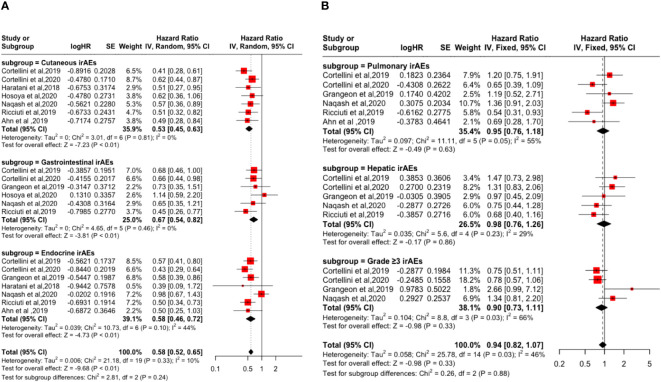
Forest plot of the association between the occurrences of different irAEs types and grades and PFS. **(A)** Cutaneous irAEs; Gastrointestinal irAEs; Endocrine irAEs; **(B)** Pulmonary irAEs; Hepatic irAEs; Grade ≥3 irAEs.

**Figure 4:**
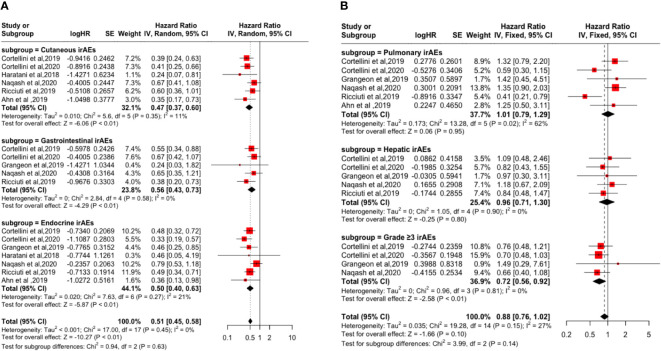
Forest plot of the association between the occurrences of different irAEs types and grades and OS. **(A)** Cutaneous irAEs; Gastrointestinal irAEs; Endocrine irAEs; **(B)** Pulmonary irAEs; Hepatic irAEs; Grade ≥3 irAEs.

Subgroup analysis based on irAEs types and grades showed that cutaneous irAE [PFS: (HR: 0.53, 95% CI: 0.45-0.63, P <0.01); OS: (HR: 0.47, 95% CI: 0.37-0.60, P <0.01)], gastrointestinal irAE [PFS: (HR=0.67, 95% CI=0.54-0.82, P <0.01); OS: (HR: 0.56, 95% CI: 0.43-0.73, P <0.01)], endocrine irAE [PFS: (HR: 0.58, 95% CI: 0.46-0.72, P <0.01); the OS: (HR: 0.50, 95% CI: 0.40-0.63, P <0.01)], and grade ≥3 irAEs [PFS: (HR: 0.90, 95% CI: 0.73-1.11, P = 0.33); OS: (HR: 0.72, 95% CI: 0.56-0.92, P <0.01)] is a favorable factor for the improvement of PFS and OS. However, pulmonary irAE [PFS: (HR: 0.95, 95% CI: 0.76-1.18, P = 0.63); OS: (HR: 1.01, 95% CI: 0.79-1.29, P = 0.95)] and hepatic irAE [PFS: (HR: 0.98, 95% CI: 0.76-1.26, P = 0.86); the OS: (HR: 0.96, 95% CI: 0.71-1.30, P = 0.80)] happened not improvement factor of PFS and OS (p > 0.05). As shown in **Figures 3** and **4**.

### Sensitivity analysis and publication bias

In the sensitivity analysis, the results of OS and PFS remained significant regardless of which study was deleted, indicating that the significant associations between the occurrence of irAEs and the response to ICIs and survival outcomes in NSCLC patients remained stable (**Additional File 1: Supplementary Figures S1**, **S2**). In the meta-analysis, funnel plots and Begg tests were used to assess publication bias (32). As can be seen from the funnel plot, the symmetrical spread of the effect points of the independent studies and the Begg test showed no significant asymmetry for PFS (p = 0.058) (**Attached File 1: Supplementary Figure S3**). For OS, the funnel plot shows a symmetrical spread of the independent study effect points, and the Begg test also shows no significant asymmetry for OS (p = 1.000) (**Supplementary 1: Supplementary Figure S4**).

## Discussion

Although the underlying pathophysiology has not been explicitly articulated to date, there is growing evidence that the occurrence of irAEs is an independent predictor of NSCLC patients receiving immunotherapy. This study provides a more comprehensive and extensive analysis of the relationship between irAEs and patient survival outcomes. In our analysis, we found that the presence of irAEs was a favorable factor for the survival prognosis of patients. Possible explanations are that irAEs are caused by overactivation of autoreactive T cells and that patients who respond to ICIs are at greater risk of developing irAEs. Stratified analysis based on irAEs type showed that cutaneous, gastrointestinal, and endocrine irAEs were favorable factors for the improvement of OS and PFS (P<0.05). However, no significant association was found between hepatobiliary irAEs, pulmonary irAEs, and favorable outcomes. The possible reason is that adverse events in the liver, lung, and other important organs can lead to irreversible damage to their function, and they cannot tolerate other anti-tumor therapy, thus affecting the prognosis. In contrast, adverse events related to the cutaneous, gastrointestinal tract, and endocrine system are relatively easier to control, which also leads to a difference in the occurrence site and prognosis of irAEs.

Although in this study, the presence of grade 3 or above irAEs showed a good correlation with survival outcomes, which is inconsistent with previous studies. The authors suggest that there was no significant correlation between the occurrence of grade 3 or higher irAEs and good survival outcomes. First of all, we went back to the original text and found that in the Cortellini et al. (19, 20) study, the gastrointestinal tract was the most common type of grade 3 or above irAEs, followed by hepatopulmonary irAEs, cutaneous irAEs, and endocrine irAEs. However, if the above high-grade irAEs are included in the analysis simultaneously without subgroup analysis for different types of high-grade irAEs, the adverse effects of hepatopulmonary irAEs on survival outcomes are likely to be masked by cutaneous, gastrointestinal, and endocrine irAEs with better prognosis, which may lead to bias in the final results. It even produces a better prognosis. Therefore, more studies are needed in the future to conduct subgroup analyses of high-level irAEs to confirm this problem. Second, according to the guidelines, the occurrence of grade 3 irAEs requires the suspension or permanent discontinuation of ICIs therapy, which will eventually lead to disease progression and affect survival outcomes. However, there are still differences between the organs of grade 3 or higher irAEs and the prognosis; for example, except for grade ≥3 bullous dermatitis, Stevens-Johnson syndrome, and toxic epidermal necrolysis requiring permanent disuse of ICIs, most of the other types of irAEs, such as rashes and pruritus, can be relieved or cured after local or systemic steroid treatment. Endocrine-related irAEs can also continue ICIs therapy after receiving alternative therapy or symptomatic therapy. The main manifestations of gastrointestinal irAEs are diarrhea or colitis, both of which can be well controlled by hormone therapy. However, checkpoint inhibitor pneumonitis (CIP), once detected, requires immediate suspension or discontinuation of ICIs and symptomatic treatment. In addition, the occurrence of CIP is closely related to PD-L1 and programmed death-ligand 2 (PD-L2), and studies have shown that PD-L1 and PD-L2 have important but opposite roles in regulating airway hyper reactivity (AHR) and invariant natural killer T (iNKT) cell-mediated activation and maintaining internal environment stability. Under normal circumstances, the interaction of the two can inhibit the inflammatory response of T helper 2 (Th2) cells, and when ICIs disrupt this balance, it can lead to CIP (33, 34). Direct inhibition of PD-1 also increases the likelihood of increased toxicity (35). The reason for the poor prognosis of CIP may be due to the fact that CIP can appear in grades ≥3 irAEs in the early stages of the disease, and the disease progresses more rapidly (36). Therefore, this relationship leads us to realize that the absence of adverse events after ICIs treatment may indicate a lack of efficacy. On the contrary, because different types of adverse events have different pathophysiological mechanisms, the response to ICIs and the degree of damage to the body are also different. Patients with pulmonary, hepatogenic, and high-grade adverse events often have a poor prognosis, possibly due to the need to discontinue ICIs after irAEs, combined with organ system damage that prevents further antitumor therapy in the short term and ultimately leads to disease progression. Therefore, not all irAEs can improve the prognosis of patients. Close attention should be paid to the occurrence of pulmonary, hepatogenic, and high-grade adverse events, and identification and active treatment should be carried out as early as possible to effectively control the progression of the disease.

This study was subject to several limitations inherent in the study design and the included studies. First, we included HR reported in the study rather than individual patient data. In addition, synthetic analyses of OS and PFS showed significant heterogeneity, which may be due to different types and grades of irAEs. Although subgroup analysis of irAEs was performed in this study to reduce the influence of heterogeneity, cutaneous, gastrointestinal, and endocrine-related adverse events were more common in irAEs, and the prognosis was good, while liver and lung irAEs showed poor prognosis. In the analysis of irAEs grade, if the type of irAEs includes liver and lung irAEs, the study results may be overshadowed by irAEs such as cutaneous with a better prognosis. Therefore, future research needs to further investigate this issue. However, despite these limitations, we provide a meta-analysis of irAEs versus survival outcomes in patients with NSCLC, and irAEs can serve as a promising prognostic biomarker in patients with NSCLC.

## Conclusion

In conclusion, the available evidence suggests that irAEs may be a prognostic biomarker for patients with NSCLC. However, further research is needed to explore the prospect of irAEs as a prognostic biomarker for patients on immunocombination therapy.

## Data availability statement

The original contributions presented in the study are included in the article/**Supplementary Material**. Further inquiries can be directed to the corresponding author.

## Author contributions

SM: Conceptualization, Funding acquisition, Investigation, Methodology, Software, Visualization, Writing – original draft, Writing – review & editing. HN: Data curation, Formal analysis, Investigation, Methodology, Validation, Writing – original draft. CW: Formal analysis, Investigation, Methodology, Writing – original draft. CJ: Investigation, Methodology, Writing – original draft. LW: Conceptualization, Data curation, Investigation, Methodology, Project administration, Resources, Supervision, Writing – review & editing.
