# Supplementary material for: Association between immune-related adverse events and prognosis in patients with advanced non-small cell lung cancer: a systematic review and meta-analysis
Source: Front Oncol. 2024 May 8;14:1402017. doi: 10.3389/fonc.2024.1402017 (PMC11109391; doi:10.3389/fonc.2024.1402017)
Supplement: Supplementary file 1 [file DataSheet_1.docx]

**
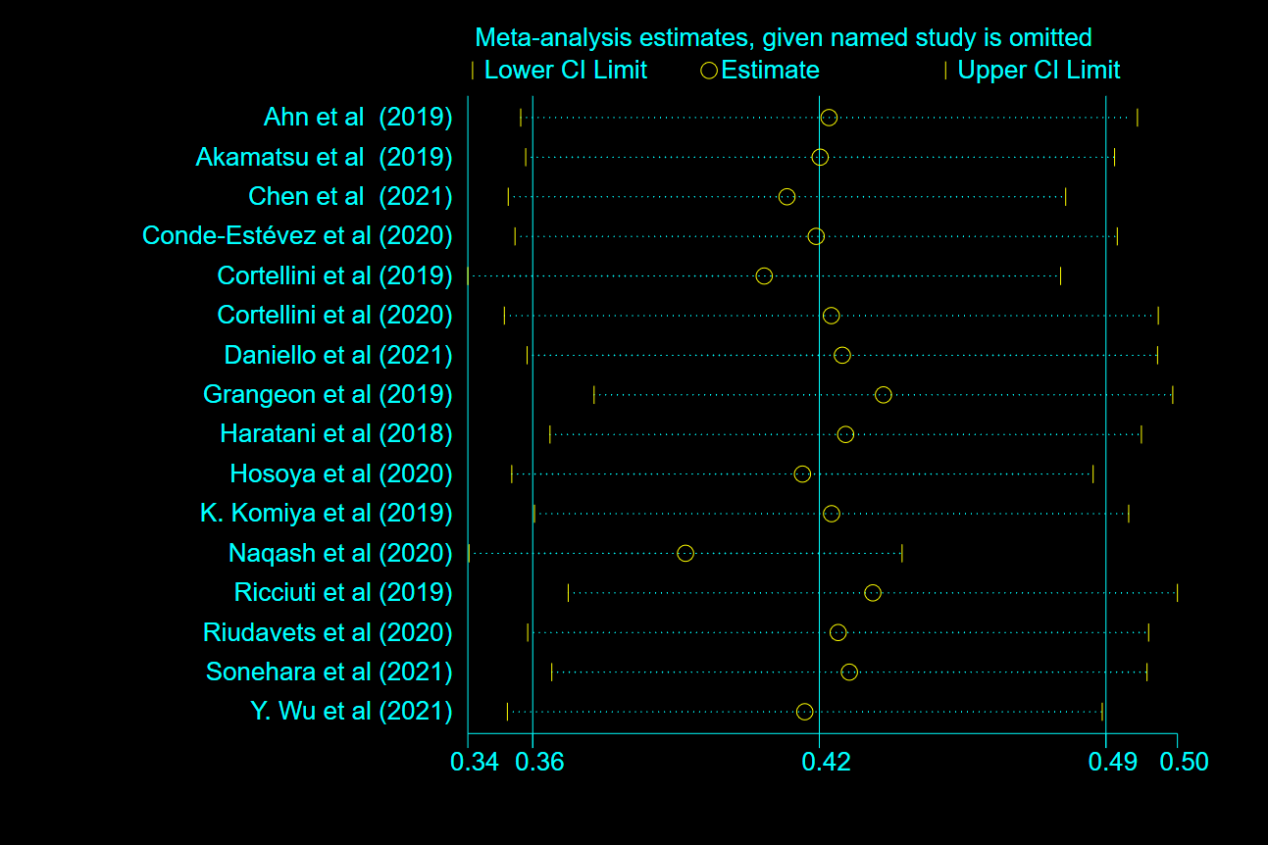
**

**Figure S1. Sensitivity analysis of OS.**

**
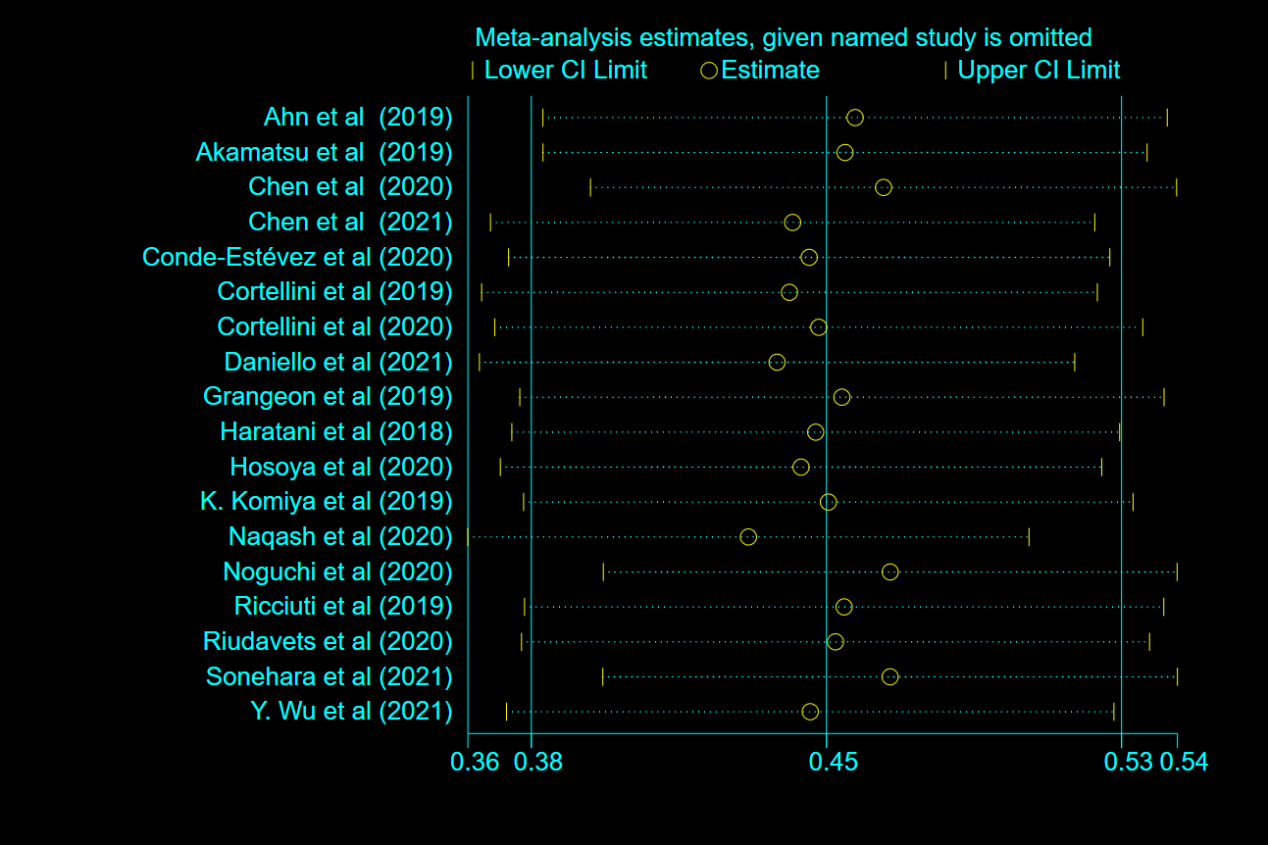
**

**Figure S2. Sensitivity analysis of PFS.**

**
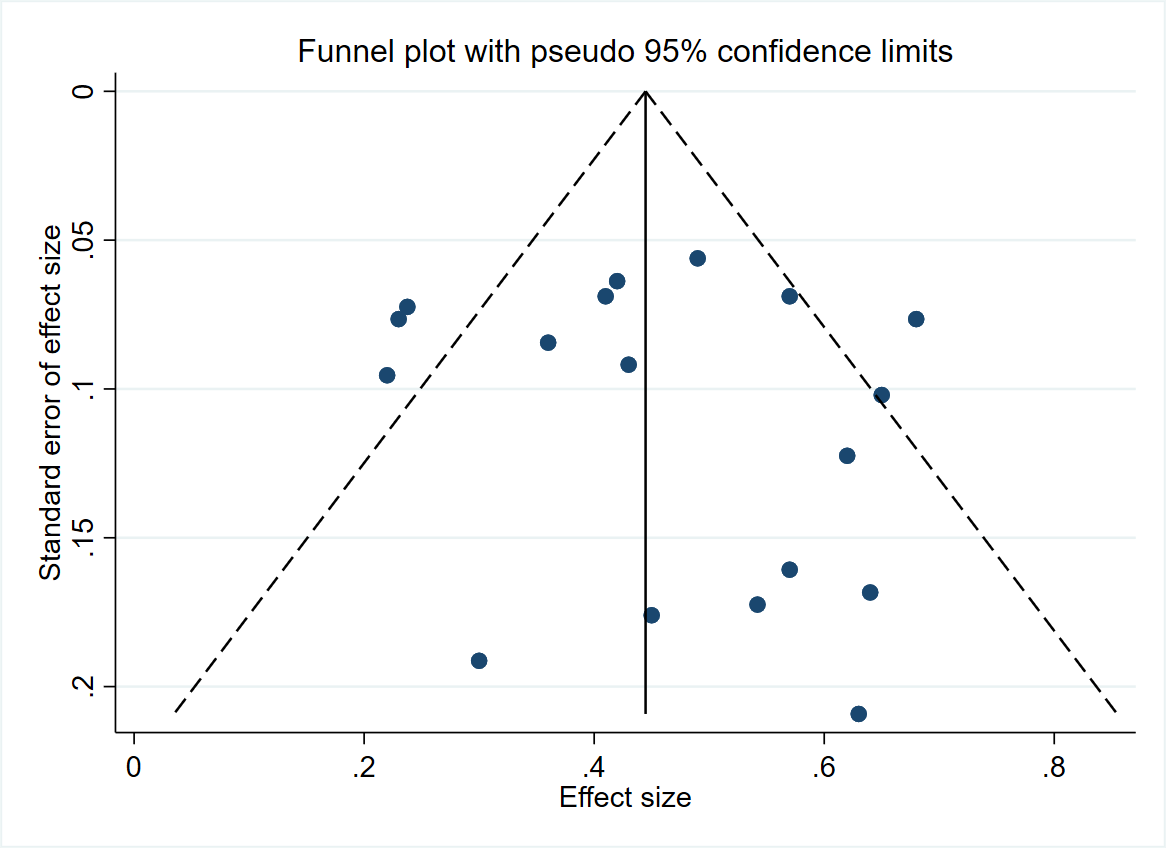
**

**Figure S3. Funnel plot for the publication bias of PFS.**

**
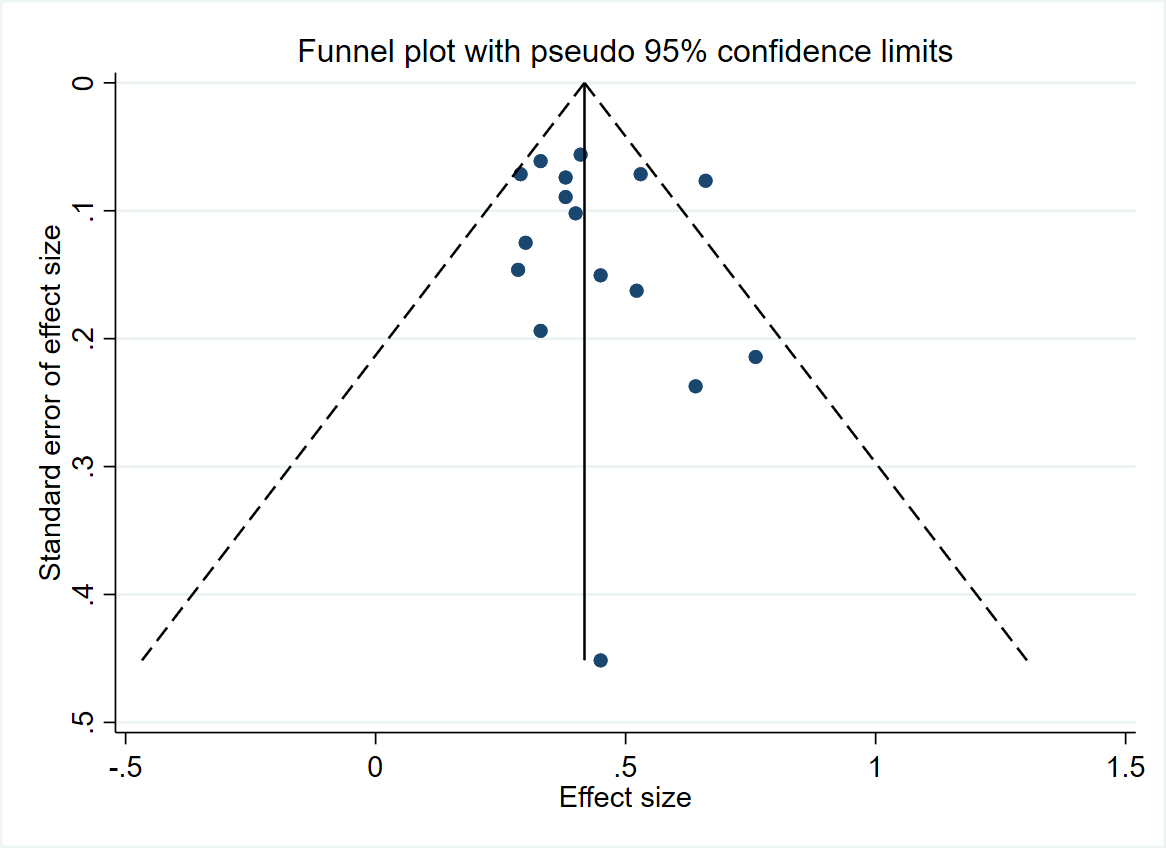
**

**Figure S4. Funnel plot for the publication bias of OS.**
